# A Cheerful Report from Ipswich

**Published:** 1921-09-10

**Authors:** 


### A Cheerful Report from Ipswich.

A good idea of the position of the East Suffolk and Ipswich Hospital and the policy of its management may be gleaned from the first paragraph of the annual report, which reads as follows:

"Looking back over the past fiscal year, we realise that it has been characterised by many anxieties and perplexities, relieved by the achievement of some of our cherished purposes and brightened by cordial support from every quarter. We started the year embarrassed by a debt of over £10,000, conscious of the fact that the income was not sufficient to meet current expenses, sympathetically aware of the suffering of 250 people who were awaiting admission to the hospital and for whom we had no accommodation, alarmed at the decline in the value of securities, and apprehensive of jeopardising the financial status of the institution by plunging into increased expenditure consequent upon additional accommodation.

"We closed the year rejoicing that the bold policy which we adopted had been fully justified, and we look forward to the consummation of our schemes of extension and improvement in the near future."

Another large temporary ward has been added to the equipped beds during the year, bringing the total number up to 300. No fixed charge has been made for patients, but each one has been asked to contribute what he or she can reasonably afford, and a sum of £3,120 collected by this means. The debt of £10,000 was paid by a fête which raised £2,400, and by a grant of £8,600 from the National Relief Fund. All previous debit balances and deficiencies have been liquidated, and the only overdrawn account is that of the year's work, which shows a deficiency of £2,000, a state of finance with which the management is naturally gratified.

The large scheme of permanent extensions, for which the sum of £50,000 has already been raised, has not been started owing to the difficulties in the building trade, but the work will be put in hand so soon as conditions improve. The hospital is maintaining a temporary Convalescent Home at Felixstowe, pending the erection of a new permanent building on a valuable site which has been acquired. The accounts show that there is a capital sum available under the will of the late Dr. J. H. Bartlet, a former President of the hospital, for the purpose of the building and endowment of the new Convalescent Home, amounting to £130,000.

The report is made by Mr. J. D. Cobbold, who has been Chairman of the Institution for the past seven years, and who terminates his report with the following: "Our Secretary, Mr. Arthur Griffiths, has shown at all times and in every direction conspicuous ability in conducting the affairs of the hospital."

The hospital has decided not to alter the date of its financial year, which ends, as hitherto, on May 31. [When the new local committee gets to work may not this matter be again reconsidered?—Editor.]

